# Dissemination of VIM-2 producing *Pseudomonas aeruginosa* ST233 at tertiary care hospitals in Egypt

**DOI:** 10.1186/s12879-015-0861-8

**Published:** 2015-03-12

**Authors:** Mai Mahmoud Zafer, Mohamed Hamed Al-Agamy, Hadir Ahmed El-Mahallawy, Magdy Aly Amin, Seif El Din Ashour

**Affiliations:** Department of Microbiology and Immunology, Faculty of Pharmacy, Ahram Canadian University, 4th Industrial Zone, Banks Complex، 6th of October, Giza, Egypt; Department of Pharmaceutics and Microbiology, College of Pharmacy, King Saud University, 11451 Riyadh, Saudi Arabia; Department of Microbiology and Immunology, Faculty of Pharmacy, Al-Azhar University, Cairo, Egypt; Department of Clinical Pathology, National Cancer Institute, Cairo University, Cairo, Egypt; Department of Microbiology and Immunology, Faculty of pharmacy, Cairo University, El Aini, As Sayedah Zeinab, Cairo, Egypt

**Keywords:** *Pseudomonas aeruginosa*, Carbapenem resistance, *bla*_VIM-2_, ST233, Egypt

## Abstract

**Background:**

*Pseudomonas aeruginosa* is an important nosocomial pathogen, commonly causing infections in immunocompromised patients. The aim of this study was to examine the genetic relatedness of metallo-beta-lactamase (MBL) producing carbapenem resistant *Pseudomonas aeruginosa* clinical isolates collected from 2 tertiary hospitals in Cairo, Egypt using Multi Locus sequence typing (MLST).

**Methods:**

Phenotypic and genotypic detection of metallo-beta-lactamase for forty eight non-duplicate carbapenem resistant *P. aeruginosa* isolates were carried out. DNA sequencing and MLST were done.

**Results:**

The *bla*_VIM-2_ gene was highly prevalent (28/33 strains, 85%) among 33 MBL-positive *P.aeruginosa* isolates. MLST revealed eleven distinct Sequence Types (STs). A unique ST233 clone producing VIM-2 was documented by MLST in *P.aeruginosa* strains isolated from Cairo university hospitals. The high prevalence of VIM-2 producers was not due to the spread of a single clone.

**Conclusions:**

The findings of the present study clearly demonstrate that clones of VIM-2 positive in our hospitals are different from those reported from European studies. Prevalence of VIM-2 producers of the same clone was detected from surgical specimens whereas oncology related specimens were showing diverse clones.

## Background

*Pseudomonas aeruginosa*, an opportunistic pathogen, is an important cause of infection in patients with impaired immune systems [1]. *P. aeruginosa* is a prime example of a species that has continually evolved such that many strains are extensively drug resistant—i.e., resistant to all standard anti-pseudomonal antibiotics (carbapenems and aminoglycosides) and sensitive only to colistin [2]. Nowadays, intensive clinical use of carbapenems has caused the presence of carbapenem resistant *P. aeruginosa* populations [3] and an increase in carbapenem resistance by acquisition of different mechanisms, such as hyperproduction of chromosomal AmpC beta-lactamase, overexpression of efflux systems, alteration or lack of outer membrane proteins (such as porin OprD), and production of carbapenemases [4]. The advent of mobile MBL genes heralded a new chapter in resistance; class 1 integrons, the mobile genetic structures that carry MBL genes, also carry genes encoding determinants of resistance to aminoglycosides and other antibiotics, and thus confer extensive drug resistance [5]. MBL gene *bla*_VIM-2_ was first reported in *P. aeruginosa* in France in 2000, but the earliest recorded case was in Portugal in 1995 [6]. VIM-2 has emerged as a dominant MBL variant worldwide. MultiLocus sequence typing (MLST) has identified international clonal complexes (CCs) responsible for the dissemination of MBL-producing *P.aeruginosa*, particularly in European countries [7], in Japan [8], Singapore, and Brazil.

The aim of this study was to examine the genetic relatedness of MBL producing *P. aeruginosa* strains isolated from two hospitals in Cairo, Egypt.

## Methods

### Bacterial isolates

Forty eight non-duplicate carbapenem resistant *P. aeruginosa* isolates were obtained from clinical specimens submitted for bacteriological testing from hospitalized in-patients admitted to Kasr Al Aini Hospital (KAA) and National Cancer Institute (NCI), Cairo University, Egypt from January 2011 to January 2012. KAA School of Medicine and NCI are tertiary hospitals belonging to Cairo University, Egypt. The study was approved by the Ethics Committee of Cairo University and an informed consent was obtained from all patients receiving treatment and participating in the study.

### Isolate identification

Initial identification and susceptibility testing was done using VITEK 2 (bioMerieux, Marcy l’E’toile, France) automated machine. Genotypic identification was carried out by PCR amplification and sequence determination of 16S rDNA previously described by Spilker *et al.* [9]. The identified strains were stored in glycerol broth cultures at - 70°C.

### Antimicrobial susceptibility testing

Susceptibility testing was done by the disc diffusion method of the Clinical and Laboratory Standards Institute, [10] with discs from Oxoid (Oxoid ltd., Basin Stoke, Hants, England). MICs were determined with E-test strips (bioMerieux, Marcy L’Etoile, France). *P. aeruginosa* ATCC 27853 was used as a control throughout.

### Phenotypic detection of MBLs

E-test MBL strips were in accordance with the manufacturer’s instructions to seek MBL production. A no less than eight-fold reduction in imipenem MIC in the presence of EDTA, or a phantom zone, was taken as a positive result.

### Detection of MBL-encoding genes and integrase genes

*bla*_*VIM-2*_*, bla*_*IMP-1*_*, bla*_*SIM*_*, bla*_*GIM*_*, bla*_*SPM*_*, bla*_*NDM-2*_ and *intI1* were amplified for *P.aeruginosa* isolates using primers listed in Table 1 according to the previous protocols [11-15]. Negative and positive controls were involved in all PCR experiments. Five microliters of reaction mix containing PCR product were analysed by electrophoresis in 0.8% (w/v) agarose (Fermentas, Lithuania).

**Table 1:** **List of primers used in this study**

**Primers**	**Sequence 5′-3′**	**Use**	**Expected PCR product**	**Reference**
**PA-SS-F**	GGGGGATCTTCGGACCTCA	Identification	956 bp	Spilker *et al.* [9]
**PA-SS-R**	TCCTTAGAGTGCCCACCCG			
***bla***_**IMP-1**_	TGAGCAAGTTATCTGTATTC	*bla*_IMP-1_	740 bp	Yan *et al*. [11]
	TTAGTTGCTTGGTTTTGATG	amplification		
**VIM-F1**	ATGTTCAAACTTTTGAGTAAGTTATT	*bla*_VIM-2_	801 bp	Frasson *et al*. [12]
**VIM-FK**	ATGTTAAAAGTTATTAGTAGTTTATTGKTC	amplification and sequencing		
**VIMR1**	CTACTCGGCGACTGAGC			
**VIMR2**	CTACTCAACGACTGAGCGA			
***bla***_***NDM***_		*bla*_*NDM*_	984 bp	Kaase *et al.* [13]
_**PreNDM-A**_	CACCTCATGTTTGAATTCGCC	amplification		
_**Pre-NDM-B**_	CTCTGTCACATCGAAATCGC			
***bla***_**GIM**_	TCGACACACCTT GGT CTG AA	*bla*_GIM_	477 bp	Ellington *et al.* [12]
	AACTTCCAACTT TGCCATGC	amplification		
***bla***_**SPM**_	AAAATCTGGGTACGCAAA CG	*bla*_SPM_	271 bp	Ellington *et al.* [12]
	ACATTATCCGCTGGAACAGG	amplification		
***bla***_**SIM**_	TAC AAG GGATTCGGCATCG	*bla*_SIM_	570 bp	Ellington *et al.* [12]
	TAATGG CCTGT CCCATG TG	amplification		
***intI1***		*intI1*	457 bp	Shibata *et al*. [15]
**Int-1-F**	GCA TCC TCG GTT TTC TGG	amplification and sequencing		
**Int-1-R**	GGT GTG GCG GGC TTC GTG			

### DNA sequencing

Amplified products of *bla*_VIM-2_ were purified using a QIAquick PCR Purification Kit (Qiagen, Crawley, UK) and sequenced in both directions using the ABI Prism 3700 DNA Sequencer (Applied Biosystems, Foster City, CA). The types of β-lactamase genes were identified by comparison with the sequences in GenBank (http://blast.ncbi.nlm.nih.gov/Blast.cgi).

### Multi-locus sequence typing (MLST)

PCR and sequencing of the recognized chromosomal markers (7 housekeeping genes) *acs*A, *aro*E, *gua*A, *mut*L, *nuo*D, *pps*A, and *trp*E were done [16]. The nucleotide sequences of these genes were compared with the sequences submitted to the MLST database to determine the allelic numbers and sequence types.

The collection of MLST data on *P. aeruginosa* is available on http://pubmlst.org/paeruginosa/. MLST was performed for twenty isolates, representatives of the *bla*_VIM-2_ gene identified. All these isolates were imipenem resistant, MBL producing and VIM-2 positive. An isolate from each hospital was selected randomly for comparison.

## Results

Of the 48 carbapenem resistant *P.aeruginosa* isolates, 33 (68.7%) were confirmed to be MBL producers. Of the 48 *P.aeruginosa* isolates, 21 isolates were from wound, 12 were from urine, 9 were from sputum,4 were from blood and two were from ear swab; 16 (48.5%), 8 (24.2%), 6 (18.1%), 2 (6.1%) and 1 (3.0%) produced MBL, respectively.

Each of MBL positive strains and their MIC results are summarized in Table 2.

**Table 2:** **Isolation details, VIM-2 gene results, MICs and sequence type of 33** ***P.aeruginosa*** **MBL positive isolates**

**Isolate**	**Duration of episode in days**	**Sample**	**Hospital**	**VIM-2**	**NDM-1**	**IMP-1**	**PM**	**PTc**	**TZ**	**TZL**	**CI**	**AK**	**GM**	**IP**	**CT**	**Sequence type (ST)**
1	21	Wound	KAA	+	-	-	≥256	16	≥256	≥256	2	12	3	≥32	≥256	
2	9	Blood	KAA	+	-	-	12	64	8	3	≥32	16	≥256	≥32	≥256	
3	36	Wound	KAA	+	+	-	8	64	64	24	≥32	≥256	12	≥32	≥256	233
4	30	Wound	KAA	+	-	-	4	3	6	2	≥32	≥256	≥256	≥32	32	233
5	13	Wound	KAA	+	-	-	4	3	6	1.5	≥32	≥256	≥256	≥32	32	
6	8	Wound	KAA	+	+	-	64	≥256	≥256	≥256	≥32	≥256	≥256	≥32	≥256	233
7	5	Wound	KAA	+	-	-	24	≥256	≥256	192	≥32	≥256	12	≥32	≥256	
8	10	Wound	KAA	+	-	-	6	96	96	24	0.094	4	2	≥32	≥256	
9	10	Urine	KAA	+	-	-	≥256	3	≥256	≥256	≥32	32	12	≥32	≥256	303
10	9	Blood	KAA	+	-	-	2	4	1.5	≥32	2	2	≥32	16	≥256	
11	75	Sputum	KAA	+	-	-	≥256	≥256	≥256	≥256	≥32	≥256	32	≥32	≥256	
12	4	Wound	KAA	+	-	-	6	24	8	16	≥32	≥256	1.5	≥32	≥256	198
13	30	Urine	NCI	+	-	-	≥256	≥256	≥256	≥256	≥32	≥256	≥256	≥32	≥256	629
14	10	Sputum	NCI	**-**	-	-	≥256	24	≥256	≥256	1	6	2	≥32	≥256	
15	30	Wound	NCI	**-**	-	+	16	24	16	4	≥32	12	≥256	≥32	≥256	
16	46	Wound	NCI	+	-	-	≥256	≥256	48	8	≥32	96	≥256	≥32	≥256	233
17	22	Sputum	NCI	+	-	-	6	12	12	2	≥32	6	≥256	≥32	≥256	507
18	70	Sputum	NCI	**-**	-	-	≥256	4	≥256	≥256	≥32	96	32	≥32	≥256	
19	65	Sputum	NCI	+	-	-	≥256	≥256	≥256	96	0.094	96	32	≥32	≥256	406
20	120	Sputum	NCI	+	-	-	64	32	12	2	≥32	16	≥256	≥32	≥256	303
21	54	Wound	KAA	+	-	-	8	16	24	4	≥32	48	≥256	≥32	≥256	233
22	27	Wound	KAA	+	-	-	16	≥256	≥256	≥256	≥32	128	32	≥32	≥256	274
23	7	wound	KAA	+	-	-	256	≥256	≥56	256	≥32	≥256	32	≥32	≥256	884
24	45	Urine	KAA	+	-	-	8	12	≥256	4	0.064	8	≥256	≥32	≥256	738
25	150	urine	KAA	+	-	-	≥256	≥256	≥256	128	≥32	48	32	≥32	≥256	274
26	75	Wound	KAA	+	-	-	≥256	≥256	≥256	≥256	≥32	≥256	≥256	≥32	≥256	683
27	21	Urine	KAA	+	-	-	16	≥256	≥256	32	0.064	8	32	≥32	≥256	990
28	45	Urine	KAA	**-**	-	-	64	≥256	≥256	≥256	0.064	32	32	≥32	≥256	
29	240	Urine	KAA	+	-	-	2	4	12	2	0.125	4	2	≥32	≥256	233
30	180	Urine	KAA	+	-	-	2	4	8	1.5	0.125	4	2	≥32	≥256	233
31	21	Wound	KAA	+	-	-	8	128	6	24	0.094	6	2	≥32	≥256	
32	30	Wound	KAA	**-**	-	-	12	32	2	8	0.064	6	2	≥32	≥256	
33	21	Ear swab	KAA	+	-	-	6	6	≥256	8	3	≥32	128	16	≥32	233

PM = cefepime, PTc = piperacillin/tazobactam, TZL = ceftazidime/clavulanic acid, TZ = ceftazidime, CI = ciprofloxacin, AK = amikacin, GM = gentamicin, IP = imipenem, CT = cefotaxime.

Of the 33 MBL isolates, MBL encoding genes *bla*VIM was identified in 28(85%) isolates, *bla*NDM was identified in 2 (6.1%) isolates and *bla*IMP was identified in only one (3.0%) isolate. In this work MBL *bla*GIM, *bla*SIM, and *bla*SPM allele were not detected. Twenty nine (87.8%) of the MBL-producing isolates were positive for class 1 integron. All the VIM-2 producing isolates had the class 1 integron. All isolates were resistant to imipenem (MIC ≥ 8 μg/ml). Sequencing of intrinsic *bla*VIM confirmed that the nucleotide sequences obtained were identical to genes for VIM-2 for *P.aeruginosa*. Similarly, the nucleotide sequences of class І integron also coincided with the results predicted by the PCR analyses. Eleven distinct STs were identified. Results of STs analyzed are recorded in Table 2. Antibiotic susceptibility result for 33 MBL producing *P.aeruginosa* is given in Table 3.

**Table 3:** **Resistance patterns of 33** ***P. aeruginosa*** **MBL positive strains**

**Isolate**	**Type of specimen**	**Antibiotic disk diffusion susceptibility test**														
		**Imipenem**	**Augmentin**	**Cefuroxime**	**Cefoperzone**	**Ceftazidime**	**Ciprofloxacin**	**Meropenem**	**Amikacin**	**Sulperazone**	**Levofloxacine**	**Gentamicin**	**Norfloxacine**	**Tazocin**	**Ceftriaxone**	**Polymixin B**
1	Wound	R	R	R	R	R	R	R	R	R	R	R	R	R	R	S
2	Blood	R	R	R	R	R	R	R	R	I	R	R	R	R	R	S
3	Wound	R	R	R	R	R	R	R	R	R	R	R	R	R	R	S
4	Wound	R	R	R	R	R	S	R	R	R	R	R	S	R	R	S
5	Wound	R	R	R	R	R	R	R	R	R	R	R	R	R	R	S
6	Wound	R	R	R	R	R	S	R	R	R	R	R	R	S	R	S
7	Wound	R	R	R	R	R	R	R	R	R	R	R	R	R	R	S
8	Wound	R	R	R	R	R	S	R	R	R	R	R	R	R	R	S
9	Urine	R	R	R	R	R	R	R	R	R	R	R	R	R	R	S
10	Blood	R	R	R	R	R	R	R	S	R	R	S	R	R	R	S
11	Sputum	I	R	R	R	R	I	R	R	R	R	R	R	I	R	S
12	Wound	R	R	R	R	R	R	R	R	R	R	R	R	R	R	S
13	Urine	R	R	R	R	R	R	R	S	R	R	R	R	R	R	S
14	Sputum	R	R	R	R	R	I	R	S	R	R	S	R	S	R	S
15	Wound	R	R	R	R	R	R	R	S	S	R	R	R	R	R	R
16	Wound	R	R	R	R	R	R	R	R	R	R	R	R	R	R	S
17	Sputum	R	S	R	R	S	R	R	R	R	R	R	R	R	R	S
18	Sputum	R	R	R	S	S	R	R	R	R	R	R	R	R	S	S
19	Sputum	R	R	R	R	R	S	R	S	R	R	S	R	R	R	S
20	Sputum	R	R	R	R	R	R	R	R	R	R	R	R	R	R	S
21	Wound	R	R	R	R	R	R	R	R	R	R	R	R	R	R	S
22	Wound	R	R	R	R	R	S	R	R	R	S	R	R	R	R	S
23	wound	R	R	R	R	R	S	R	R	R	S	R	R	R	R	S
24	Urine	R	R	R	R	R	R	R	R	R	R	S	R	R	R	S
25	Urine	R	R	R	R	R	I	R	R	R	R	R	R	R	R	S
26	Wound	R	R	R	R	R	I	R	R	R	R	R	R	R	R	S
27	Urine	R	R	R	R	R	R	R	R	R	R	R	R	S	R	S
28	Urine	R	R	R	R	R	S	R	R	R	S	R	S	R	R	S
29	Urine	R	R	R	R	R	S	R	R	R	R	R	R	R	R	S
30	Urine	R	R	R	R	R	R	R	R	R	R	R	R	R	R	S
31	Wound	R	R	R	R	R	S	R	R	R	S	R	R	R	R	S
32	Wound	R	R	R	R	R	R	R	R	R	R	S	R	S	R	R
33	Ear swab	R	R	R	R	R	R	R	S	R	R	R	R	R	R	S

## Discussion

This study clearly demonstrates that carbapenem non-susceptible *P. aeruginosa* is present in Egypt in isolates of different origins mainly isolated from surgical wards with high prevalence of VIM-2 positivity in MBL producing *P.aeruginosa* isolates. The ability of MBL-producing *P. aeruginosa* to reach high level endemicity in certain settings has, indeed, been established, [5] and in these cases, MBLs can outpace loss of oprD and up-regulated efflux pumps as leading factors causing reduced carbapenem susceptibility; distinct clones even unfolding their potential for causing outbreaks [17]. Our results revealed that 33 (68.7%) of 48 carbapenem resistant *P.aeruginosa* isolates produced MBL. This study demonstrated an increasing prevalence of MBL. Similarly high prevalence of MBL producing *P.aeruginosa* was detected in the Egyptian study where 82% were MBL producers [18]. In another Egyptian study 32.3% MBL producing *P.aeruginosa* was observed which is lower than our findings [19]. In the present study, VIM-2 was the most frequently detectable gene among the different MBL genes investigated; the percent of 85% among carbapenem-resistant MBL producing *P. aeruginosa* was detected. This finding was supported by results of previous studies demonstrating VIM-2 as the most dominant MBL implicated in imipenem resistant *P.aeruginosa* and confers the greatest clinical threat [20]. Worldwide, VIM-2 is the dominant MBL gene associated with nosocomial outbreaks due to MBL-producing *P. aeruginosa* [21]. Since MBL-producing isolates can cause serious infections that are difficult to treat, their presence in various hospitals in Egypt is of nationwide concern. The absence of new agents for the treatment of infections caused by these bacteria may lead to treatment failures with increased morbidity and mortality. A class 1 integron carries the integrase gene (intI1), which encodes the site-specific recombinase responsible for cassette insertion. In our study class 1 integron was confirmed in 29 (87.8%) of MBL producing *P.aeruginosa*. This result is consistent with findings from Malaysia in which they suggested that the class 1 integron is the most abundant type of integron present among the clinical isolates of *P.aeruginosa* in Malaysia [22].

MDR *P.aeruginosa* isolates, resistant to almost all β-lactams, aminoglycosides and quinolones, often ascribed to epidemic clones (ST235 or ST111), have been detected in hospitals worldwide, mainly within ICU [23]. The increasing prevalence of MDR *P. aeruginosa* isolates is a global health problem, because of the limitation in clinical treatment options. All the VIM-producing isolates in this study belonged to international clones. In this study the results of MLST showed that VIM-2 type MBL carbapenem resistant *P. aeruginosa* specimens isolated from surgical wards of Kasr Al Aini hospital showed similarity in which ST233 which was a part of the internationally dominant clonal cluster CC233 was detected in 7/15 isolates indicating health care acquired transfer of *P.aeruginosa* could occur and should be prevented, an increased risk of cross-transmission and high antimicrobial pressure might have favoured clonal spread. Additionally, patients who have the potential to facilitate dissemination of MDR organisms between hospitals subsequently, might serve as important reservoirs and transmission sources, stressing the importance of hand hygiene compliance, and patient precautions, whereas diversity in sequence types is shown from specimens isolated from NCI where 5 distinct sequence types were observed. This diversity could be due to previous empirical intake of antibiotics or misuse. ST 233 VIM-2 producing *P.aeruginosa* was detected in one isolate imported from Ghana in a study done in Norway and Sweden in which the two international clonal complexes CC111 and CC235 associated with MBL-producing *P. aeruginosa* isolates were dominant [24]. In previous studies from different Russian states, VIM-2 positive ST 235 was the predominant isolated type that has rapidly spread throughout Russia, Belarus and Kazakhastan via clonal dissemination. The authors were not able to prove the reason for spread of VIM-2 positive ST 235 but explained that inappropriate use of antibiotics and poor adherence to infection control practice could be among the reasons [25]. Although *P.aeruginosa* clinical isolates are seldom typed and therefore under-reported, ST235 association with VIM-2 seems Europe centered, and has been reported in Belgium, Croatia, Serbia, and, more recently, Greece [26]. In other parts of Europe ST 111 were the major sequence type detected in MBL producing *P.aeruginosa* and ST 446 was detected but less widespread [27]. Thus it seems that different clones could be detected from different geographical areas.

## Conclusions

The findings of the present study clearly demonstrate that clones of VIM-2 positive in our hospitals are different from those reported from European studies as none of our isolates revealed ST 235. Prevalence of VIM-2 producers of the same clone was detected from surgical specimens whereas oncology related specimens were showing diverse clones. Similar ST seems to be transmitted due to poor adherence to infection control policies which necessitate efforts for more strict abidance to infection control regulations. Different clones from different specialty hospitals in our study requiring further investigations to explain the relation of diversity of ST types to cause of resistance.
